# Role of Adenotonsillectomy and Tonsillectomy in Children with Down Syndrome Who Develop Obstructive Sleep Apnea by Obesity as a Risk Factor

**DOI:** 10.1155/2022/8074094

**Published:** 2022-05-06

**Authors:** Imran Ali Khan

**Affiliations:** Department of Clinical Laboratory Sciences, College of Applied Medical Sciences, King Saud University, Riyadh, Saudi Arabia

## Abstract

Down syndrome (DS) or trisomy 21 is caused due to the presence of additional chromosome 21 in humans. DS can exist either as free trisomy 21 (nondisjunction), Robertsonian translocated DS, or as mosaic DS. Obstructive sleep apnea (OSA) is a complex condition with serious health implications for pediatric individuals with DS. OSA is common in DS, and when it is present, it appears to be extreme. Obesity and snoring are some of the OSA risk factors for children associated with DS and OSA. Adenotonsillectomy is one of the surgical protocols applied in children, which is useful in lowering the OSA in which obesity is commonly connected within normal and DS children. Tonsillectomy is the alternative procedure of surgery connected with postoperative respiratory complications, and adenotonsillectomy was found to be a safe surgical method in children and improves the quality of life. The main aim of this review is to bridge the gap between the role of OSA in normal children (46, XX/XY) and DS children (47, XX/XY+21) characterized by the presence of chromosomes and exactly what is the involvement with adenotonsillectomy and tonsillectomy when obesity is a risk factor. The treatment for OSA and obesity is rehabilitative and reversible; however, DS can be managed but not resolved because the disorder occurs from the existence of an extra chromosome during the failure of homologous chromosomal pairing separation during maternal meiosis I. This review concludes that there is a treatment for OSA and obesity and that DS children can be prevented from being obese or experiencing OSA but cannot be turned to normal chromosomes due to an extra trisomy 21. According to this review, children with DS and OSA/OSAS, as well as concomitant complications, can be treated.

## 1. Introduction

Down syndrome (DS; OMIM 190685) is a genetic disorder of trisomy 21 (G and mongolism) with the frequency of 1/700 births [1]. The most common genetic cause of mental retardation in humans is trisomy 21 and is one of the aneuploidy defects caused by live-born infants [2]. Among trisomy 21, 2 copies of chromosome 21 will be inherited from the mother and 1 copy from the father. The mother produces a couple of chromosome copies due to the failure of separation of homologous chromosome pairing during maternal meiosis I [3]. The extra copy of 21 human chromosome (HSA21) is classified as free trisomy: i.e., 47, XY/XX, +21, and this is the most frequent DS type. These specific types of trisomies occur due to the premature separation of sister chromatids during the first meiotic division [4]. The presence of trisomy 21 thus changes the development of the embryo and causes genetic characteristics associated with DS. The bonus chromosome triggers developmental delays, as well as mental and physical disability in children with DS [5]. The diagnosis of DS is based on clinical features and cytogenetic analysis and fluorescent in situ hybridization technique [6]. DS children are more likely to have obstructive sleep apnea (OSA) than normal children. Some of the physical traits are low muscle tone, small stature, an upward slant to the eyes, and a single deep crease across the center of the palm—although no one is exactly alike [7]. In addition, genetic and environmental influences were proposed to interactively lead to aneuploidization, including dietary considerations. Birth prevalence is correlated to maternal age, which is a major risk factor for trisomy 21. The unique phenotype associated with DS is through numerous congenital anomalies such as gastrointestinal tract, tracheoesophageal fistula, pyloric stenosis, imperforate anus, and Hirschsprung disease [8].

Most forms with free trisomy 21 (85-90%) are the product of maternal meiosis errors. The most impaired stage of the nondisjunction is maternal meiosis I (>75%) in particular, whereas the errors of maternal meiosis II constitute >20%. In 5% of maternal and paternal free trisomy 21 chromosomes, paternal meiotic errors can be seen; here, meiosis II nondisjunction happens at a greater frequency than I error. Postzygotic mitotic errors (5%) have also been identified in humans [9]. Despite that such an occurrence can occur during the development of either the egg or the sperm, it is likely a maternal origin event, occurring during the first meiotic division in the maturing oocyte; as a result, only less than 10% of primary trisomy 21 occurs of paternal origin [10].

**Figure 1:**
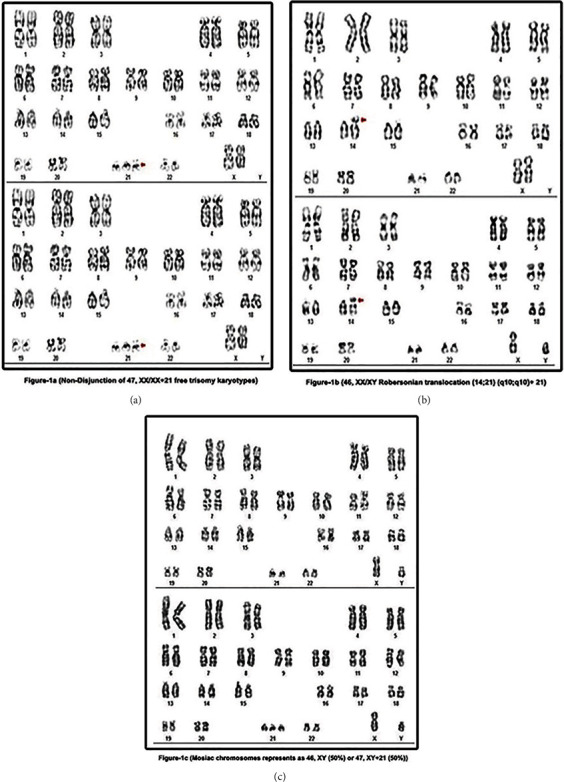
Representation of (a) free trisomy 21, (b) Robertsonian translocated DS, and (c) mosaic DS with 46/47+21 XX and XY chromosomes.

**Figure 2:**
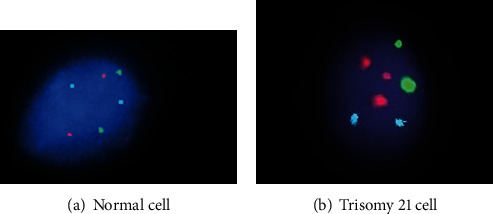
Representation of trisomy 21 through fluorescent in situ hybridization technique. (a) Normal cell showing the signal pattern 2 red, 2 green, and 2 blue and (b) abnormal cell showing the signal pattern 3 red, 2 green, and 2 blue indicating trisomy 21 (Down syndrome).

About 92.5% have normal trisomy 21, although 4.8% of extra chromosome 21 content is found in the form of an unbalanced Robertsonian translocation or as an isochromosome of the long arm of chromosome 21. The remaining 2.7% are heterogeneous and contain mosaic, double trisomy and inverse translocation. DS are categorized into nondisjunction (95%), translocation (3%), and mosaicism (2%) (Table 1 and Figure 1). However, Figure 2 represents trisomy 21 through fluorescent in situ hybridization technique.

Nondisjunction (free trisomy 21) is defined as the occurrence of error in the human cells which result in triplets in the chromosome, i.e., presence of 3 copies of chromosomes instead of 2 copies. During or at the time of conception, either the sperm or the egg cannot divide a pair of 21^st^ chromosomes. The extra chromosome 21 is reproduced in each cell of the body as the fetus develops trisomy 21 (47 XX+21 and 47XY+21). The transfer of genetic material between the couple of chromosomes is defined as translocation and exchange of genetic material between chromosomes 14 and 21 is defined as Robertsonian translocation of DS which occurs within the acrocentric chromosomes, i.e., 13-15 and 21-22. The appearance of the same individuals of more than two separate cell lines is called mosaicism which could be germline mutation, somatic mutation, or both. DS is a somatic mutation that occurs in non-Mendelian heritage mosaicism or mosaic DS [6].

## 2. Maternal Age and Down Syndrome

**Figure 3:**
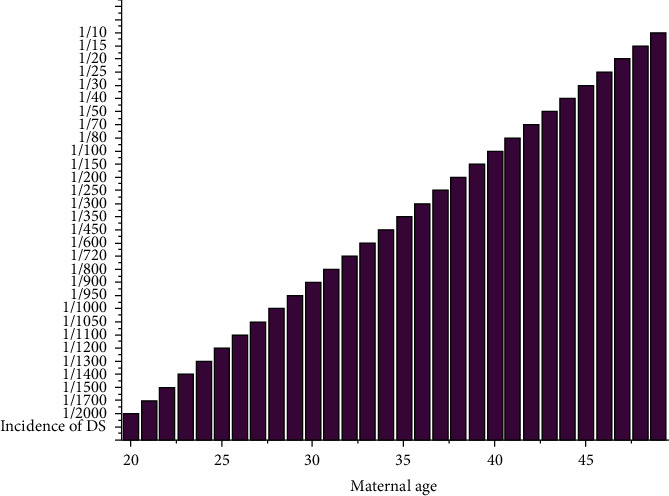
Incidence of Down syndrome in relation to maternal age.

Clinical entity may be associated with chromosome 21 trisomy and their results with ageing. The factor of maternal age (<35 years of age) still dominates the etiology of DS and tends to arise from natural causes regardless of geographic or socioeconomic distinction. A major risk factor cannot be clearly ascertained in the disease etiology because the documentation is ambiguous. There is no compelling case for a paternal age impact for trisomic proabortuses. Pairing of heteromorphic chromosome bands showed that fathers contribute extra chromosome in 15-20% of DS children, and this approach is only informative in 30-60% of cases.  Figure 3 describes that the relation between prevalence of DS is connected to the maternal age.

## 3. Maternal Obesity and Its Relation to Down Syndrome

Maternal obesity is associated with congenital birth defects; high maternal age and obese pregnant women are at increased risk for gestational diabetes and a poor socioeconomic status [11]. Maternal obesity is transferred to infant obesity during the first trimester of pregnancy. Additionally, birth weight is also linked up with the BMI later in life. The transfer of relative risk of infant obesity correlating with maternal obesity was between 2.0- and 2.3-folds in 2-4 years of children [12]. A study documents that children diagnosed with DS tend to become either overweight or obese in the future. However, the prevalence was found to be low when compared with the general population. The prevalence of obesity in DS was documented to be between 33 and 71% [13].

## 4. Purpose of This Review

The main purpose of this review is to bridge the gap between the role of OSA in normal children (46, XX/XY) and DS children (47, XX/XY+21) characterized by the presence of chromosomes and exactly what is the involvement with adenotonsillectomy and tonsillectomy when obesity is a risk factor.

## 5. Obstructive Sleep Apnea and Its Relation to Down Syndrome

Obstructive sleep apnea or OSA is a general disease characterized by frequent episodes of partial or total obstruction of the respiratory tract during sleep. OSA is commonly identified in children, and parents often pursue medical treatment for their children who snore, breathe through their mouths, or have OSA which is a common sleep-related breathing disorder that is potentially lethal, including intermittent narrowing of the neck airway during sleep [14]. An apnea is traditionally described as breathing cessation for >10 s, but like most other terms, this is quite arbitrary. Gastaut et al. [15] defined three different types of apneas: (i) obstructive apnea occurs as air pressure stops but rotation of the chest wall (rib cage and abdomen) continues, implying breathing effort in the presence of a blocked upper airway; (ii) central apnea, in which all flow and movement halts, presumably due to a loss of the drive to breathe; and (iii) apnea mixed is a variation of the above two patterns, with characteristics indicating an initial core and then obstructive incidents [16]. Children with severe OSA may develop congestive heart failure and cor pulmonale [17]. The obstructive sleep apnea syndrome (OSAS) is represented as one of the breathing disorders during sleep which is characterized through prolonged partial airway or intermittent complete obstructions which interrupts normal ventilation during various patterns of sleep. For detecting DS children with OSA, a parent history is more likely to have an accurate diagnosis. Children with OSAS were diagnosed mostly by cardiologists and endocrinologists in the 1950s when they approached with heart failure or severe growth impairment. Cardiovascular complications such as pulmonary hypertension, cor pulmonale, and heart failure were formerly thought to be the most common forms of this syndrome in children [17]. The association of lymphoid tissue in the naso- and oropharynx with OSA is considered in DS, as is an integrated approach to pharyngeal and palatal surgery, which leads to routine sleep. Children with DS are also vulnerable to general hypoventilation, and severe sleep disorders in DS children are extremely uncommon [18]. DS children would have an infinite number of predisposing factors for the formation of OSAS such as midfacial and mandibular hypoplasia, glossoptosis, a superficially located tonsil and relative tonsil, adenoidal invasion of the superficial upper airways, elevated secretions, a rise in the prevalence of lower respiratory tract anomalies, obesity, and generalized hypotonia that causes airways to collapse during inspiration. At the time, a maxillofacial CT scan revealed bilateral bony choanal atresia as well as a very shallow nasopharynx with skull base apposition near to the atretic plates [19]. Subsequently, individuals with DS have endocrine, anatomic, biochemical, and OSAS [20]. Genetic risk was correlated with independent risk factors such as obesity, maternal smoking, and premature birth. The prevalence of OSA in the general pediatric population is 0–5.7% [21], and in DS, the prevalence ranges from 45 to 76% [22]. OSAS has a 30-60% prevalence in DS children when compared in 2% of healthy children.

Common symptoms, physical examinations, and differential diagnosis of OSA in children have been tabulated in Table 2. Adenotonsillar hypertrophy (ATH) and lingual tonsils were commonly documented between the physical examinations and differential diagnosis of children with OSA [23]. ATH is one of the most common causes of OSA in children. OSA risk factors include ATH, craniofacial anomalies, obesity, and neuromuscular disorders. The most common cause of upper airway obstruction in children is ATH, and adenotonsillectomy is the primary surgical treatment for pediatric patients with OSA [24]. Obesity is one of the key risk factors for OSA and is the only one that can be modified and is irreversible [25].

Obesity is well linked to chronic sleep loss, erratic lifestyle, and a significant pathogenic factor for OSA [26]. Previous studies have documented that breathing sleep disorder is more prevalent in obese than in nonobese children. OSA can also be exacerbated by being underweight. However, the pathophysiology is still elusive. The children without obesity could have greater adenotonsillar hypertrophy than obese children, and in obese children, there could be a lower severity of adenotonsillar hypertrophy [27]. With increased obesity, sleep apnea may lead to the development of daytime alveolar hypoventilation, cor pulmonale, and frank respiratory failure. OSA is connected to DS through obesity and mainly through the maternal age and nondisjunction. Obesity in pregnant women is influenced by a rapid increase in vascular complications linked to pregnancy such as preeclampsia, pregnancy-induced hypertension, and gestational diabetes. Various sleep disorders affect children with DS; specifically, sleep disordered breathing and OSA are common among DS. Nerfeldt et al.'s studies conclude that the prevalence of OSA in DS children was over 80%. Children with DS have numerous malformations, medical complications, and cognitive disability due to inheriting extra genetic material from chromosome 21 known as trisomy 21 [7]. The pediatric population with DS will be affected by congenital heart defects, thyroid disease, and sleep disorders [28]. Epidemiological studies have shown that individuals with DS can develop OSA at a rate of 50-100% in children and 100% in adults [29–33]. Additionally, Maris et al.'s [30] study concluded as 66% of subjects developed OSA in DS subjects [34]. The American Academy of Pediatrics recommends adenotonsillectomy as frontline treatment for OSA children and adenotonsillar hypertrophy. Maris et al. [35] confirmed that adenotonsillectomy results showed significant OSA in DS children without altering the sleep patterns and severe OSA was associated with a more significant reduction in the severity. A similar statement was released by Nation's [36] studies documented through his systemic review.

## 6. Treatment

**Figure 4:**
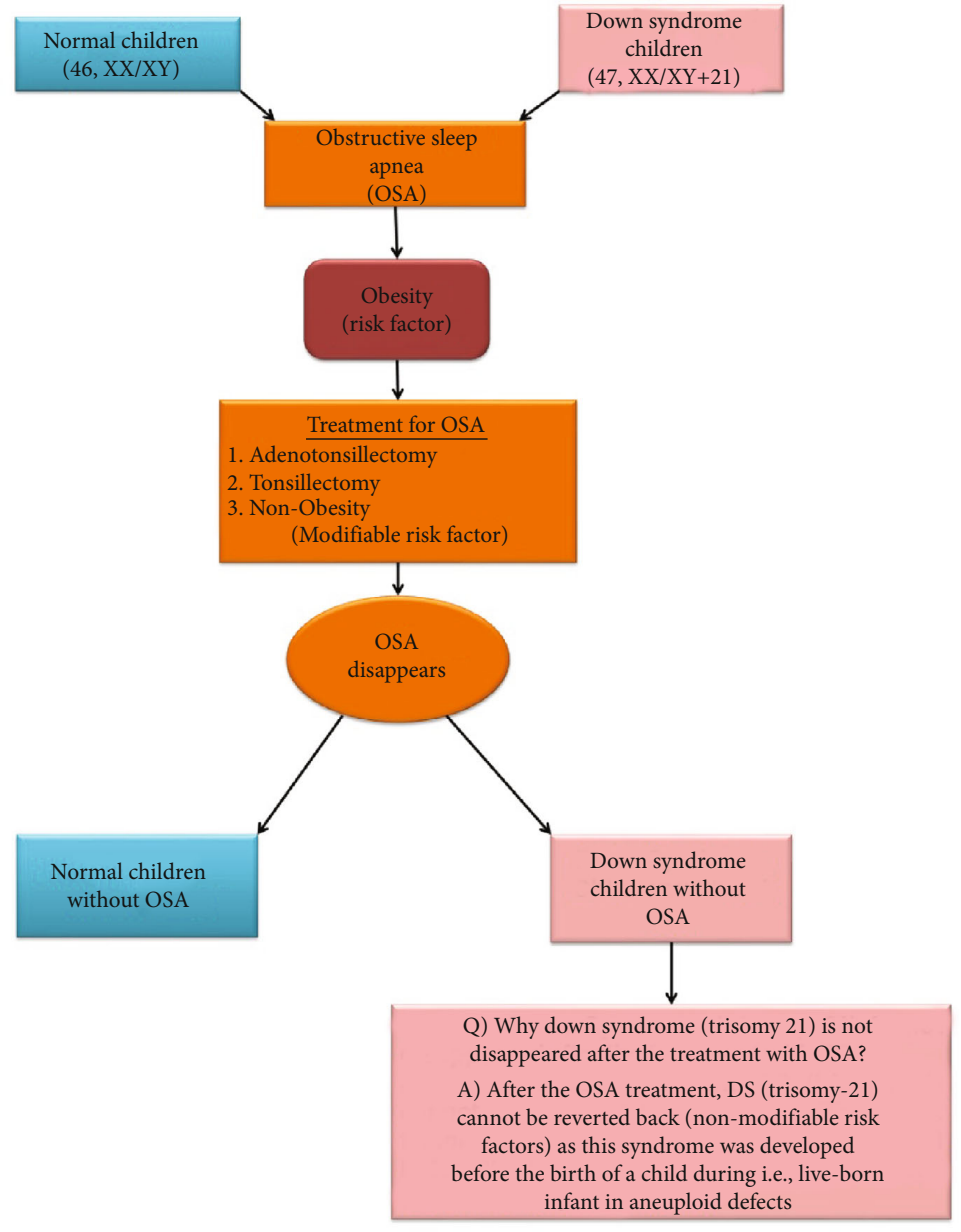
Diagrammatic representation of development of OSA and its treatment.

In pediatric population with critical OSA and adenotonsillar hypertrophy, tonsillectomy with adenectomy is the first-line surgery. It also demonstrated a significant improvement in apnea-hypopnea index incidence, oxyhemoglobin saturation, and sleep quality in obese OSA patients. Positive airway pressure is regarded as the gold standard treatment for patients suffering from OSA. The effectiveness of CPAP therapy is restricted by patient therapy enforcement. The study of the oral cavity and oropharynx provides insight into the protentional upper airway surgery. Nasal obstruction has been recognized as a key factor in the treatment of OSA [37]. Adenotonsillectomy surgery is one of the most commonly performed procedures on children, which complains of adenotonsillar disorders. Recurrent tonsillitis and OSA are the two most common reasons for adenotonsillectomy, and it is a surgical procedure that removes the adenoids as well as the tonsils. Adenotonsillectomy is the standard first-line treatment for pediatric SDB, but it may not be curative when other risk factors, such as obesity, are present. Tonsillectomy and adenoidectomy are surgical procedures that remove the small glands on either side of the throat (tonsils) and at the top of the throat below the nose (adenoids). Tonsillectomy, with or without adenoidectomy, has long been studied and is one of the most commonly performed surgical procedures in the pediatric age group around the world [38, 39]. The success rate of adenotonsillectomy treatment in otherwise stable, nonobese children is around 80%. Tonsillectomy is the alternative procedure of surgery, and body mass index is correlated in numeral ways. However, this method has a lot of postoperative respiratory complications. So, adenotonsillectomy was found to be the safe surgical method in children [40]. Bower [41] studies documented that 69% of symptoms disappeared with the surgical treatment of tonsillectomy and adenoidectomy in DS patients. DS is connected with obesity which tends to develop OSA and using surgical treatment with adenotonsillectomy, proves to lower the symptoms of OSA effectively, and improves the quality of life. The medical treatment of adenotonsillectomy can also be avoided if the children tend to lose the extra weight. Based on the treatments with both adenotonsillectomy and tonsillectomy, OSA can be cured with or without postoperative limited complications such as hemorrhage, fever, infections, and pain.  Figure 4 explains how both normal and DS children develop OSA and after the treatment how OSA disappear. Obesity is considered one of the most common criteria in both children and mother during maternity. However, after the OSA treatment, DS cannot be reverted back as this syndrome was developed before the birth of a child, i.e., a live-born infant with aneuploid defects. However, the query is still raised whether DS children can avoid the development of OSA in the future during their adultery or not. The published studies indicate that normal children can also develop OSAS with or without obesity. The final confirmation is that DS children can avoid obesity and OSA but DS children cannot be reverted to normal children as DS children have additional trisomy 21.

## 7. Conclusion

DS is one of the aberrant chromosomal disorders' effects in newborn children. DS children with obesity have more chances of developing OSA, and combinations of DS, obesity, and OSA can lead to cardio, neurological, and additional complications. However, normal children can also develop OSA. Previous studies documented the success of surgical treatment in adenotonsillectomy and tonsillectomy. There is no surgical or any other alternative treatment or medication for reverting the additional trisomy chromosome in DS. Obesity management is recommended for future studies because children with DS have lower basal metabolic rates than normal children. Future studies should also focus on how the clinicians can avoid developing OSA in DS children by performing the prospective randomized trial of adenotonsillectomy. This review recommends that children with DS and OSA/OSAS combined complications can be treated.

## Figures and Tables

**Table 1:** Different origins of Down syndrome (trisomy 21).

Form of DS	Standard trisomy 21 (nondisjunction)	Robertsonian trisomy 21	Mosaic trisomy 21	Partial trisomy (21q22.3)
Frequency	95%	4%	3-5%	1%
Mechanism	Maternal meiosis 85-90% (i) Meiosis I (75%) (ii) Meiosis II (25%)	Familial 25%	Mitotic	—
Mechanism	Parental meiosis 3-5% (i) Meiosis I (25%) (ii) Meiosis II (75%)	De novo 75%	—	—
Chromosomal analysis	47 XX+21 47 XY+21	46 XX/XY Robertsonian translocation between D and G chromosomes (q10; q10)+21 46XX/XY+21, i (21) (q10)	47 XX+21 47XY+21 46 XX 46 XY	46 XX/XY, duplication (21) (q22.3)

**Table 2:** Diagnosis and differential diagnosis of obstructive sleep apnea in children.

S. no.	Symptoms	Physical examinations	Differential diagnosis
1	Snoring	Obesity	Nasoseptal obstruction
2	Pulmonary hypertension	Adenotonsillar hypertrophy	Adenotonsillar hypertrophy
3	Nighttime awakening	Growth disturbances	Macroglossia
4	Unusual daytime behavior	Lingual tonsils	Lingual tonsils
5	Poor academic performance	Craniofacial abnormalities	Hypotonic pharynx
6	Cessation of breathing	Failure to thrive	Maxillary hypoplasia
7	Cyanosis	Laryngeal pathology	Laryngeal abnormality
8	Cor pulmonale	—	Enlarged soft palate/uvula
9	Excessive daytime somnolence	—	Micrognathia
10	Gasping of air	—	—
11	Enuresis	—	—
12	Irritability	—	—
